# Conduction System Pacing: Where Are We Now?

**DOI:** 10.19102/icrm.2022.130109

**Published:** 2022-01-15

**Authors:** Imran Niazi

**Affiliations:** ^1^Aurora Cardiovascular and Thoracic Services, Aurora Sinai/Aurora St. Luke’s Medical Centers, University of Wisconsin School of Medicine and Public Health

**Keywords:** Cardiac pacing, His-bundle pacing, left bundle pacing

“Physiologic” pacing and pacing for cardiac synchronization therapy have continued to pique interest over the past year, despite the global pandemic impacting health care throughout the world.

Ever since it became apparent that right ventricular (RV) pacing could worsen left ventricular (LV) function, particularly in patients with impaired ventricular function,^1^ pacing modalities that mimicked physiology have been the subject of ongoing research. Deshmukh et al. first demonstrated permanent His-bundle pacing in patients with dilated cardiomyopathy and atrial fibrillation (but a normal QRS) destined for atrioventricular (AV) junction ablation in 2000.^2^ The skill and time required, using the available technology, prevented its clinical adoption at that time. The concomitant development of biventricular pacing for cardiac resynchronization therapy (CRT) absorbed investigators’ interest and resources for the next decade, but a less than stellar response rate and advances in lumen-less lead delivery technology evoked fresh interest in alternative pacing modalities. Building on the work of Narula^3^ and E-Sherif et al.,^4^ who had shown that high-amplitude temporary pacing in the His region could resolve left bundle (LB) branch block (LBBB), Lustgarten et al. successfully demonstrated that permanent pacing in the His-bundle region could narrow the QRS in patients with LBBB and provide resynchronization equivalent to CRT.^5^

## Permanent His-bundle pacing

Permanent His-bundle pacing is inherently challenging. The bundle of His is a small target, 1–2 mm wide and 10–20 mm long, and usually encased in a fibrous insulating sheath that requires the pacing lead helix to be fixated within the structure itself or at least in its very close vicinity. Pacing thresholds are generally high and increase with time, and loss of capture over time is not uncommon. Damage from lead fixation resulting in complete heart block is not unknown. Atrial oversensing is also common, and R-waves are generally diminutive. In patients with conduction system disease and cardiomyopathy, the presence of delay in the conduction system distal to the pacing site is present in many, perhaps most, patients. Lustgarten et al. were able to show narrowing (but not normalization) of the QRS in only 72% of patients. In the His-Synch study, the only randomized prospective trial of His synchronization, the success rate of His synchronization was 56%.^6^ These reasons have prevented widespread adoption even today.

## Permanent left bundle pacing

In 2017, Huang et al. reported proximal LB pacing in a patient with LBBB and cardiomyopathy who could not undergo CRT or His synchronization. A 3830 lead (Medtronic, Minneapolis, MN, USA) was successfully screwed into the RV septum and advanced until it paced the LB, with resolution of the LB block and subsequent recovery of LV function.^7^ This was the dawn of the era of LB pacing. Huang and others went on to implant LB pacing leads in hundreds of patients, with and without conduction system disease, further refining concepts and techniques over time.

**Figure 1:**
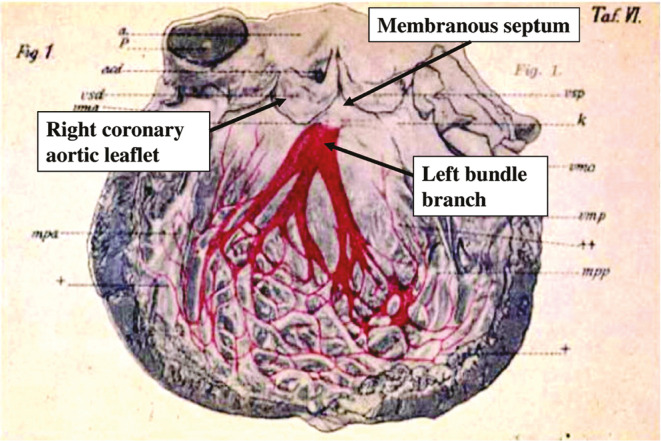
The left bundle and its branches.^10^

LB pacing is inherently simpler to perform than His-bundle pacing; the target is orders of magnitude larger **(Figure 1)**. LB pacing thresholds are low, similar to RV endocardial pacing, and do not appear to increase over time. Atrial oversensing is rare. R-waves are robust, and the lead position is generally stable. With proper technique, the 4.1-French (Fr) Medtronic 3830 lead, supported by a delivery sheath, can successfully penetrate the interventricular septum and contact the LB or its branches and arborizations.

## Clinical trials

Several investigators have reported their experience with His-bundle and LB pacing in patients with and without conduction abnormalities and with and without cardiomyopathy.

Padala et al. published the most comprehensive experience of LB pacing in (mostly) patients requiring pacing support in the absence of cardiomyopathy or bundle branch block.^8^ LB branch “area” (LBBA) pacing (LBBAP) was successful in 89% of procedures, with statistically significant narrowing of the paced QRS complex in both normal QRS and wide QRS patients. Strict criteria for LB pacing were met in only 41% of patients (the concept of LBBAP will be discussed later). At one year of follow-up, the pacing threshold and R-waves remained stable (R-wave, 15.4; threshold, 0.81; mean impedance, 582). The only complications were LB lead dislodgement in three patients. This study suggested that LBBAP was successfully accomplished in most patients with minimal complications.

Vijayaraman et al., reporting on behalf of the International LBBAP Collaborative Study Group, recently described the largest experience with LBBAP in patients with cardiomyopathy and conduction system abnormalities.^9^ The study was a retrospective analysis of data obtained at eight centers in four continents. The patient population was a heterogeneous group, with ischemic cardiomyopathy in 44% and LBBB in 39%; the remaining patients had an RV paced rhythm, right bundle branch block (RBBB), or non-specific intraventricular conduction delay.

LBBAP was successful in 277 of 325 patients (85%), with low and stable thresholds (0.6 ± 3) and R-waves (10.6 ± 6) over six months. With pacing, the QRS duration significantly decreased from 152 to 137 ms, and LV ejection fraction increased from 33% to 44%; the clinical and echocardiographic response rates were 72% and 73%, respectively. LB lead displacement occurred in 5 patients, and acute perforation into the left ventricle occurred in 10 patients; this was recognized and corrected during the procedure.

Although retrospective and observational, these data further support the thesis that this procedure is safe and probably beneficial in the right patient. They point to the need for large registries to confirm these findings and randomized controlled studies to further refine techniques and definitions.

## Clinician questions

Several clinically relevant questions concern the implanting physician: what is the most efficient implant technique in clinical practice, and how do we define successful LB pacing? To answer these, we need to recognize that implant techniques and definitions of successful LB pacing have evolved over time.

**Figure 2:**
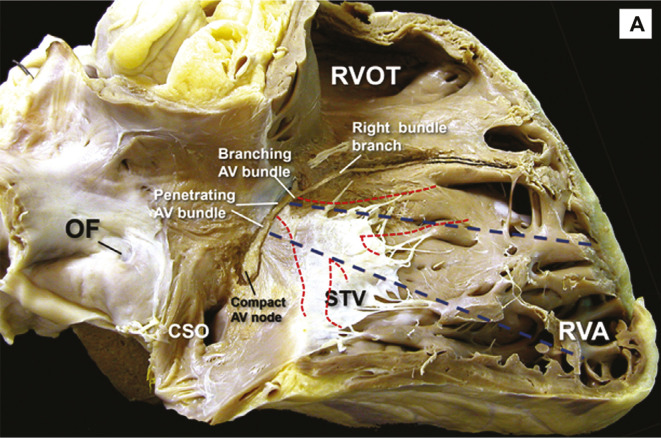
The right ventricular septum, showing the location of the left bundle and its fascicles in relation to the septal leaflet of the tricuspid valve.^8^ *Abbreviations:* AV, atrioventricular; CSO, coronary sinus orifice; OF, oval fossa; RVA, right ventricular apex; RVOT, right ventricular outflow tract; STV, septal tricuspid valve. Reprinted with permission from Cabrera J-Á, Porta-Sánchez A, Tung R, Sánchez-Quintana D. Tracking down the anatomy of the left bundle branch to optimize left bundle branch pacing. *JACC Case Rep.* 2020;2(5):750–755.

The first report by Huang et al.^7^ referred to proximal LB pacing, and the initial recommended lead position was 1.5 cm apical to the His-bundle location. The septal leaflet of the tricuspid valve impinges on the portion of the RV septum corresponding to the proximal LB, making septal penetration of the lead difficult in many cases. **Figure 2** illustrates this relationship. Indeed, the LB trifurcates at the level of the hinge of the tricuspid valve in the majority of cases.^10^ There is concern that, in this proximal location, the lead may perforate or pin the septal leaflet to the septum.

The anatomy of the human conduction system was first described by the Japanese pathologist Sunao Tawara,^9^ and **Figure 1** is an illustration from his work. It illustrates that the LB branches into 2 or 3 fascicles, which further divide and subdivide to form a complex arborizing network of even finer branches that terminate in the Purkinje fibers. This entire webwork of conducting system fibers may be thought of as the LB network; point stimulation of any branch or arborizing fiber will activate the network, its distal branches, and the LB retrogradely.

As the LB network offers a larger target than proximal LB pacing, the pacing lead may be located more apically in relation to the His-bundle location than the originally recommended distance. Lu et al. analyzed 105 successful and 93 unsuccessful LBBAP sites in 95 patients, after correcting for the RV chamber size. They found that successful pacing sites formed a cluster that was more apical in relation to the tricuspid valve than unsuccessful sites, which were more basal.^12^ Interestingly, successful sites also correlated with a leftward paced axis, consistent with a more inferior septal pacing site. It is tempting to speculate that preferential activation of the posterior fascicle is more likely to stimulate the late activated posterior lateral ventricular wall, which is a hallmark in typical LBBB, than anterior fascicle activation.

## Left bundle and left bundle area pacing

What is meant by LB branch pacing (LBBP), LBBAP, and LV septal pacing (LVSP)? What is deep septal pacing? While these terms have been used somewhat differently in older publications, some consensus appears to be emerging. LBBP is said to occur when the pacing stimulus captures the LB or its branches, with or without capture of the LV septal myocardium.^13^ If only LV septal myocardium is captured, it is referred to as LVSP or, by some, deep septal pacing. LBBAP refers to LBBP or LVSP without clear evidence for LBB capture.^14^

## Criteria for left bundle branch pacing

Early papers by Huang and others set forth several criteria for LB pacing, which included recording of an LB potential from the successful pacing site in up to 80% of cases. While this is possible in patients with intact conduction, LB potentials cannot be recorded from the pacing lead in the presence of LBBB, unless another lead is actively fixed in the His bundle and temporary His-bundle pacing is performed (pacing the His bundle with an unfixed temporary electrode requires a high-amplitude current, and the stimulation artifact obscures the LB potential). The time and radiation exposure involved render this impractical in clinical practice. Other electrophysiologic maneuvers to confirm LB capture include determination of the effective refractory period (ERP) of the LB using programmed stimulation as the LB ERP is about 30 ms shorter than that of the ventricular muscle; and measurement of retrograde stimulus–His time from the LB pacing lead (the LB–His time is shorter than the V–His time, in a manner analogous to para-His pacing for the demonstration of concealed septal bypass tracts). Again, time constraints reserve these maneuvers for the research laboratory.

Clinically useful criteria for successful LB capture include an RBBB pattern in V1, usually a QR or rSR pattern, and abrupt shortening of the interval between the stimulus artifact and the peak of the R-wave in V6 by ≥10 ms. This has been called the V6 R-wave peak time (RWPT) or, by others, the stim–LV activation time. It should be noted that LVSP (without LB capture) will also produce an RBBB pattern in V1, but the V6 RWPT will be longer than LBBP, on average by 20 ms.^15^ An LB potential can be recorded from the tip of the pacing lead in 40%–80% of patients with no conduction abnormalities and is highly specific for LBBP.

The most useful and physiologic measure of LB capture is the V6 RWPT. In patients without conduction delay, a paced V6 RWPT equivalent to the unpaced V6 RWPT confirms LB capture; normal values of 75 ms have been proposed.^16^ In patients with conduction delay, a value of 100 ms has been suggested as a good compromise between sensitivity and specificity.^15^ Absolute values may not be accurate, as conduction times can be impacted by infiltrative disease, class 1 anti-arrhythmic drugs, etc.

Measurement of V6 RWPT during LB pacing at high and low amplitudes is also useful; if V6 RWPT is shorter at high pacing amplitudes than at low amplitudes, the lead is not in contact with the LB network tissue, but it is close. The virtual cathode at high pacing amplitudes enlarges and extends to incorporate the conduction system but activates the muscle alone at low pacing amplitudes. Advancing the lead will likely attain LB capture at low pacing amplitudes. Another situation encountered during high- and low-amplitude pacing is a constant, short V6 RWPT but a slight widening of QRS at high amplitudes. This indicates selective LB capture at low amplitudes, but the extension of the virtual cathode at high amplitudes also activates the surrounding muscle.

## Clinical implications

Does LBBP provide greater clinical benefit than LVSP? Is LBBAP clinically equivalent to LBBP? Smaller studies suggest that LBBP provides better synchronization of the LV than LVSP,^17^ but large controlled clinical trials are required to answer these questions.

Can LBBAP be combined with CRT? The concept of using LV epicardial pacing in patients showing incomplete synchronization with LBBAP is appealing. The International LBBAP Collaborative Group recently published their experience^18^ and showed successful CRT and LBBAP in 81% of 112 non-consecutive patients at 8 centers. The diverse population and varied techniques do not allow firm conclusions, which will have to await randomized controlled trials.

LBBAP is in its infancy; it promises to transform pacing for both maintaining and restoring cardiac synchrony.
